# Protein Nanofibrils and Their Hydrogel Formation with Metal Ions

**DOI:** 10.1021/acsnano.0c10893

**Published:** 2021-03-05

**Authors:** Xinchen Ye, Antonio J. Capezza, Xiong Xiao, Christofer Lendel, Mikael S. Hedenqvist, Vadim G. Kessler, Richard T. Olsson

**Affiliations:** † Department of Fibre and Polymer Technology, School of Engineering Sciences in Chemistry, Biotechnology and Health, KTH Royal Institute of Technology, SE-100 44 Stockholm, Sweden; ‡ Department of Chemistry, School of Engineering Sciences in Chemistry, Biotechnology, and Health, KTH Royal Institute of Technology, Stockholm SE-100 44, Sweden; § Department of Molecular Sciences, Swedish University of Agricultural Sciences, Box 7015, 750 07 Uppsala, Sweden

**Keywords:** protein nanofibrils, whey protein, hydrogels, metal ions, kinetics

## Abstract

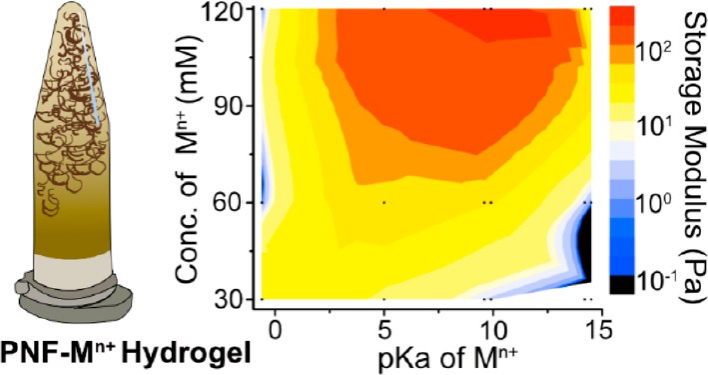

Protein nanofibrils (PNFs) have been prepared by whey protein fibrillation at low pH and in the presence of different metal ions. The effect of the metal ions was systematically studied both in terms of PNF suspension gelation behavior and fibrillation kinetics. A high valence state and a small ionic radius (*e.g*., Sn^4+^) of the metal ion resulted in the formation of hydrogels already at a metal ion concentration of 30 mM, whereas an intermediate valence state and larger ionic radius (Co^2+^, Ni^2+^, Al^3+^) resulted in the hydrogel formation occurring at 60 mM. A concentration of 120 mM of Na^+^ was needed to form a PNF hydrogel, while lower concentrations showed liquid behaviors similar to the reference PNF solution where no metal ions had been introduced. The hydrogel mechanics were investigated at steady-state conditions after 24 h of incubation/gelation, revealing that more acidic (smaller and more charged) metal ions induced *ca*. 2 orders of magnitude higher storage modulus as compared to the less acidic metal ions (with smaller charge and larger radius) for the same concentration of metal ions. The viscoelastic nature of the hydrogels was attributed to the ability of the metal ions to coordinate water molecules in the vicinity of the PNFs. The presence of metal ions in the solutions during the growth of the PNFs typically resulted in curved fibrils, whereas an upper limit of the concentration existed when oxides/hydroxides were formed, and the hydrogels lost their gel properties due to phase separation. Thioflavin T (ThT) fluorescence was used to determine the rate of the fibrillation to form 50% of the total PNFs (*t*_1/2_), which decreased from 2.3 to *ca*. 0.5 h depending on the specific metal ions added.

The preparation of protein nanofibrils (PNFs) represents an interesting field of science since it has been shown that the PNFs with outstanding mechanical properties can be made from natural resources.^1−3^ The fibrils are readily formed in nature, demonstrating not only stiffness and bending strength values of *ca*. 3.3 and 0.6 GPa, similar to conventional steel, but also biocompatibility.^4^ The possibility to produce PNFs using natural resources *in vitro*, therefore, represents an opportunity in the development of advanced materials using bottom-up chemical approaches.^5^ As liquid suspensions, PNFs frequently display strong interactions with their surroundings, resulting in the formation of hydrogels.^6−11^ It has been demonstrated that PNF hydrogels have the potential to be used in a variety of applications, for example, biomedical applications,^12,13^ optoelectronic materials,^14−16^ hybrid organic/inorganic materials,^17,18^ and as templates for the preparation of nanowires.^19−22^

The conditions under which PNFs are grown have the potential to influence the morphology of the PNFs.^23^ Affecting the fibrillation and growth may allow for the preparation of longer/thicker/curvier fibrils, which in turn have been shown to influence the macroscopic properties of PNF-based materials, resulting in, for example, stronger materials with load-bearing properties.^24,25^ The morphology of the PNFs has also been proposed to influence the rheological properties, where curved fibrils have been suggested to result in more viscous aqueous solutions by providing stronger fibril interactions in terms of entanglements.^26^ On the contrary, longer and straight PNFs have been used as skeletons to form oriented structures, for example, conductive nanowires from low viscous PNF solutions.^19,21^ The possibility of tuning the growth kinetics of PNFs and their morphology is therefore of great value since it allows for harvesting useful PNF properties in several material science application areas, such as bio-based material sensors, drug delivery, solar energy conversion, and photoluminescent materials.^27^

The synthesis of PNFs requires the unfolding of the native three-dimensional protein structures (denaturation) followed by the hydrolysis of the proteins into small peptides and the nucleation/self-assembly of the peptides to PNFs.^28−32^ To initiate and promote protein denaturation, specific conditions are used for exposing the hydrophobic segments and the hydrogen-bond donor/acceptor parts of the protein residues.^33−35^ Here, organic solvents such as urea and ethanol can effectively trigger the formation of PNFs. Another common approach is to expose the proteins to high temperatures (70–90 °C) and low pH (1.5–3.0) in aqueous solution, which denature and hydrolyze the protein.^2,36,37^ The denatured and hydrolyzed protein segments further self-assemble into PNFs, driven by a balance between electrostatic forces and hydrophobic interactions, while the unidirectional growth occurs as a consequence of repulsive forces between the fibril units.^38^ The outcome of the PNF synthesis has been shown to depend on the concentration of the starting materials and the protein source, where it has been demonstrated that protein solubility plays an important role in the fibrillation.^2,36,37,39^ Among different proteins, whey protein isolate (WPI), a side-stream product from the food industry, has been extensively studied due to its ability to form long and slender PNFs with well-defined morphologies, *ca*. 4 nm in width and up to several micrometres in length.^31,40,41^ This is in contrast to proteins generating shorter and less defined morphologies, for example, meat hemoglobin, rice globulin, and soy protein isolate.^38,39^

In this article, the PNF growth and the formation of PNF hydrogels have been studied in the presence of different metal ions, with consideration to the metal ion acidity as represented by the p*K*_a_ values ranging from 14 to −0.6 (from nonacidic to strongly acidic). A systematic selection of metal ions (Na^+^, K^+^, Co^2+^, Ni^2+^, Al^3+^, Fe^3+^, Sn^4+^, and Zr^4+^), with an increasing ability to structurally organize water as related to their increasing charge and reduction in diameter, revealed that PNF hydrogel formation occurred more readily and faster with those metal ions having low p*K*_a_ values. Previously, metal ions, for example, Na^+^, Ca^2+^, have briefly been discussed to induce changes in the fibrillation kinetics and PNF morphologies, that is, functioning as a catalyst for the PNF formation and influencing the viscosity of the PNF suspensions.^42^ It is herein demonstrated that using metal ions with different acidity is the key to forming hydrogels with different gel strength, relying on a balance between the established metal ions/water/PNF structures. Importantly, these hydrogels were formed only when the metal ions were present during the growth of the PNFs, making the interactions between the PNFs, the metal ions, and the water molecules of central importance. It is demonstrated systematically how the acidity of different metal ions affects the growth of PNFs as hydrogels, thereby allowing for designing materials that may find use in a number of future applications.

## Results and Discussion

### Metal Ion Ability to Organize PNF Solutions into Hydrogels

**Figure 1:**
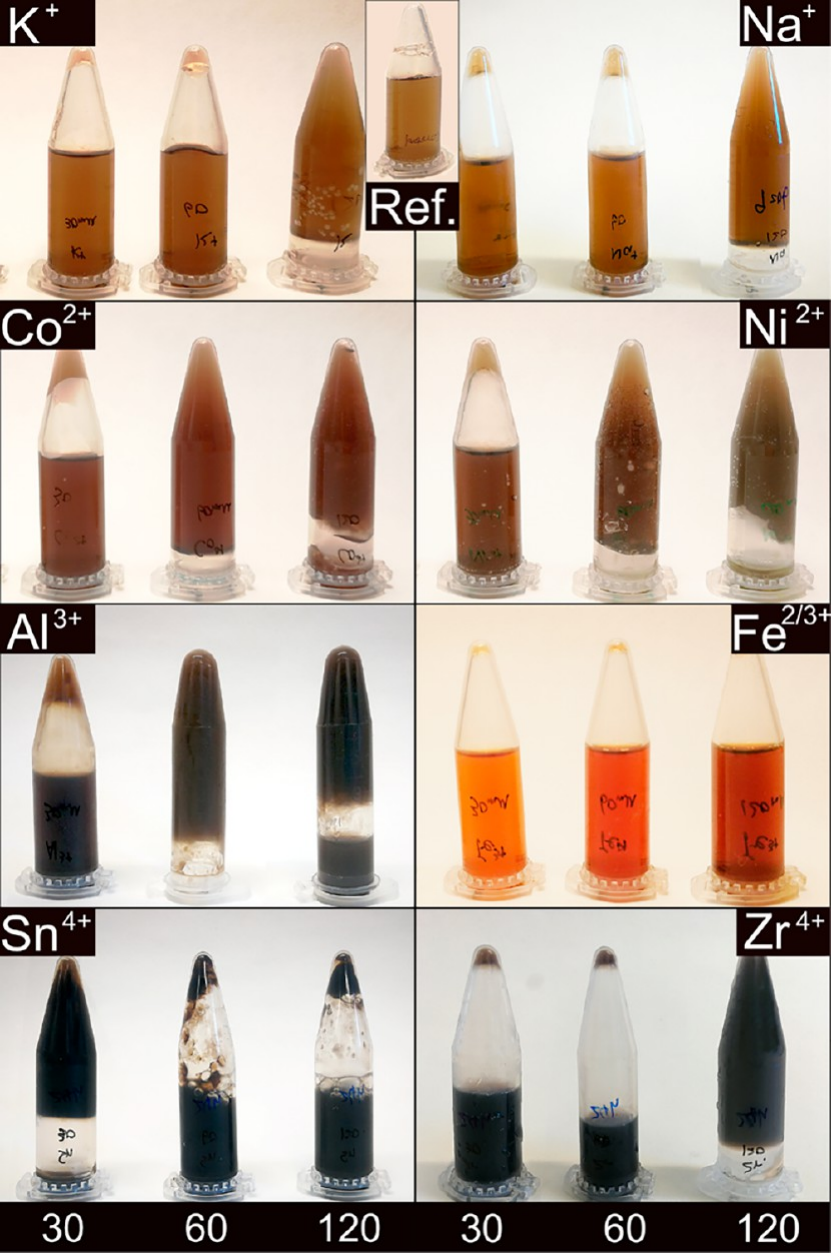
Appearance of the WPI solutions after PNF growth with and without the presence of different metal ions (at 90 °C of for 24 h). The concentrations of the metal ions were 30 mM, 60 mM, and 120 mM from left to right in each picture. Solid-phase precipitate could be identified in 120 mM Al^3+^, 60 and 120 mM Sn^4+^, and all the Zr^4+^ concentrations.

Figure 1 shows Eppendorf tubes containing PNFs grown from whey protein solutions at concentrations of 40 g/L with or without the presence of different metal ions. The tubes were standing vertically on their lids (≥10 min, steady-state) to demonstrate that at certain concentrations of the mono-, di-, tri-, and tetra-valent metal ions, an induced gelation was observed that prevented the PNF solutions from flowing in the direction of the gravitation (Figure 1). The reference sample with PNFs grown in the absence of any metal ions behaved as a low-viscous liquid and displayed no signs of gelation, see top row “Ref.” in Figure 1.

When the monovalent Na^+^ or K^+^ ions sized 116 and 152 pm, respectively, were present at a concentration of 30 mM during the growth of the nanofibrils, no difference from the reference sample was observed. A marked effect could however be seen as the concentration of Na^+^ or K^+^ ions were increased from 30 mM to 60 mM. At 60 mM, the samples transformed from low-viscous liquids to liquids with consistencies similar to syrup (not visible in the photographs). At a concentration of 120 mM, both the Na^+^ and K^+^ ions containing PNF suspensions transformed into hydrogels. These hydrogels could no longer flow as liquids to the lower section of the upside-down Eppendorf tubes, see Figure 1 top row. The 40% difference in the radius of the monovalent Na^+^ and K^+^ did not appear to make a difference for the PNF solutions behavior. It was also apparent that at concentrations ranging up to 120 mM, no color changes of the Na^+^ and K^+^ PNF-solutions occurred in comparison to the reference sample.

The second row in Figure 1 demonstrates that the divalent Co^2+^ and Ni^2+^ ions sized 88 and 83 pm, respectively, induced a strong gel formation already at a concentration of 60 mM. The Co^2+^ and Ni^2+^ metal ions also resulted in an apparent higher viscosity for the PNF suspensions containing 30 mM metal ion concentration, although the strength of these hydrogels was not enough to prevent the gels from flowing in the direction of the gravity, inside the upside-down Eppendorf tubes. The addition of even smaller sized 67 pm trivalent metal ions of aluminum, as compared to Co^2+^ and Ni^2+^, is shown in the third row (Figure 1). The Al^3+^-containing sample showed hydrogel formation at the 30 mM metal ion concentration. However, similar to Co^2+^ and Ni^2+^, the hydrogel was too weak to resist the flow in the direction of the gravity (Figure 1, Al^3+^). A firm gel was, however, again formed at 60 mM with a darker color as compared to the previously described samples (Figure 1). The Al^3+^-containing gel sample at an ion concentration of 120 mM showed an apparent phase separation, which made part of the hydrogel sediment in the Eppendorf tube. This was due to the fact that the trivalent metal ions could no longer assist in sustaining a coordination activity of water molecules and instead precipitated as amorphous aluminum hydroxide and/or oxide, see isolated particles in Figures S1c and S2.

**Figure 2:**
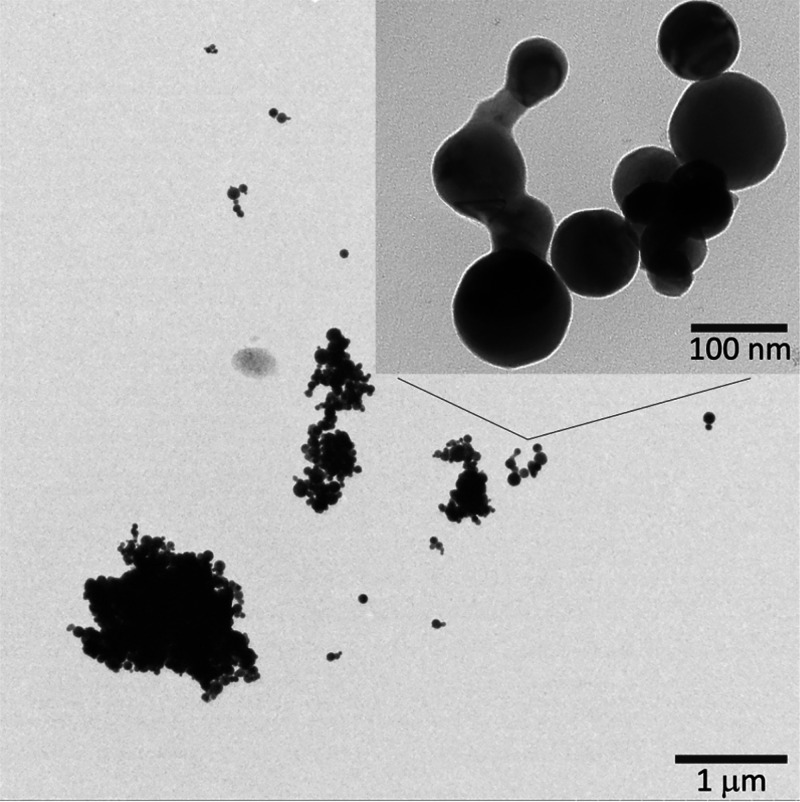
SnO_2_ nanoparticles observed by TEM in the PNF sample with 120 mM Sn^4+^.

The trend describing how more charged metal ions induce gelation at lower metal ion concentration was further confirmed by preparing a sample containing Sn^4+^ as one of the smallest metal ions herein evaluated (83 pm), with the greatest charge among all tested ions (+4). The tetravalent Sn^4+^ ion allowed formation of a firm gel at the lowest 30 mM ion concentration. The higher concentrations could however not support the formation of uniform hydrogels (in a similar fashion as for the 120 mM Al^3+^ sample), Figure 1. The precipitated SnO_2_ nanoparticles are shown in Figure 2 with their associated X-ray diffraction pattern in the supplementary section (Figure S1). The particles were spherical and showed sizes of approximately 50–200 nm, see Figure 2. It should be noted that all the X-ray diffraction patterns for the Sn^4+^ samples, regardless of the metal ion concentration, showed the crystalline peaks of SnO_2_ (Figure S1d). Even so, the sample with 30 mM ion concentration remained as a firm hydrogel and showed no phase separation in contrast to the Al^3+^ samples and the higher concentrations of the Sn^4+^ ion containing samples (Figure 1). The increase in the Sn^4+^ concentration thus proportionally affected the size and the concentration of the oxide particle formed (Figure S1d), which increased the probability of having denser particle aggregates formed for the higher concentrations. The increase in the ζ potential in the case of Al^3+^ and Sn^4+^, shown in Figure S3, also indicated the presence of the precipitated metal ion hydroxide/oxide phase for more extended incubation times, resulting in a lost ability of the metal ions to screen the protein surface charges and decrease the surface potential of the protein. However, the initial drop of the ζ potential observed for the Al^3+^ sample (Figure S3) may also be a consequence of a decrease in the double electric layer thickness of the protein molecules, possibly existing due to early interactions between Al^3+^ and the PNFs at the initial state of the incubation.

**Figure 3:**
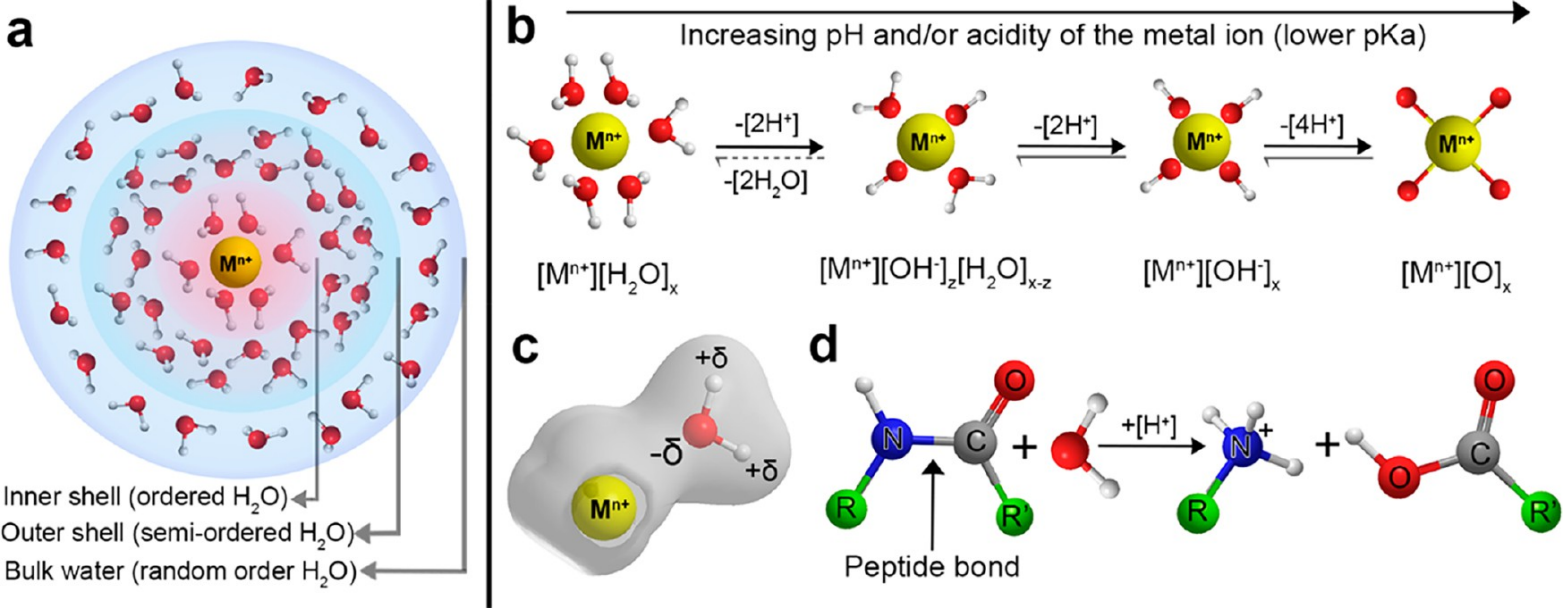
(a) Solvation shell around a metal ion, (b) hydration of metal ions, (c) polarization of water molecules, and (d) protein hydrolysis. The solvation shells in (a) illustrated as depicted also in refs (47−51).

The formation of metal hydroxides/oxides during the synthesis of the PNFs depends on the acidity of the metal ions, see Figure 3a,b. Smaller and more charged metal ions (Sn^4+^, Zr^4+^; p*K*_a_ < 0) show a greater ability in inducing hydrolysis and convert more readily the hydrated metal ions into hydroxides and oxides, which in turn do not have the same ability to coordinate water molecules in the vicinity of the PNFs. Naturally, the precipitation of the metal ions also considerably decreases the concentration of the remaining metal ions, coordinating water molecules around the growing PNFs. On the contrary, larger and less charged metal ions (Na^+^, K^+^; p*K*_a_ > 14) stay in the solution as mostly hydrated (Figure S1) but are required to be present at higher concentration to fully support a hydrogel formation caused by the metal ions coordinating the PNFs. The electronegativity (*x*_*p*_) of the metal element also needs to be considered due to its effect on the hydrolysis reactions. Table 1 shows the estimated p*K*_a_ values of the metal ions investigated in this study, as originally described by G. Wulfsberg according to eqs 1 (*x*_*p*_ < 1.5) and 2 (*x*_*p*_ > 1.5) from ref (43) :
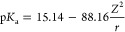
1
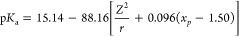
2The acidity of the PNF solutions was tested before and after the 24 h incubation to ensure the equilibrium of reaction in Figure 3b was reached, since more hydrolysis and formation of hydroxides and oxides should show as hydronium ions in the solutions (pH). Table 1 shows that the pH values of the solutions were in the range from 0.7 to 2.9 depending on the size and radius of the metal ions (in agreement with the estimated p*K*_a_ values of the metal ions, eqs 1 and 2), confirming that the smallest and most charged ions could be associated with the lowest pH values.

**Table 1:** pH and Storage Modulus of WPI Solution with and without the Presence of Metal Ions (120 mM) after 24 h Incubation at 90 °C^a^

samples	ref	K^+^	Na^+^	Mg^2+^	Ni^2+^	Co^2+^	Al^3+^	Fe^2/3+^	Zr^4+^	Sn^4+^
radius (pm)	–	152	116	86	83	88	67	92/78	86	83
p*K*_a_	–	14.5	14.2	11.4	9.9	9.6	5	9.5/2.2	–0.3	–0.6
pH	3.0	2.9	2.8	2.8	2.8	2.7	2.5	1.4	0.8	0.7
storage modulus (Pa)	0.5	21	24	182	425	155	164	–	462	0.3

a Note: The values for the radius and electronegativity were taken from refs (43−45). Storage moduli of the PNF solution/hydrogel (in the liquid state)^46^ were extracted at 1.0 Hz from the results of the frequency sweep.

### Rheology of the PNF Metal Ion/Hydroxide/Oxide Solutions

**Figure 4:**
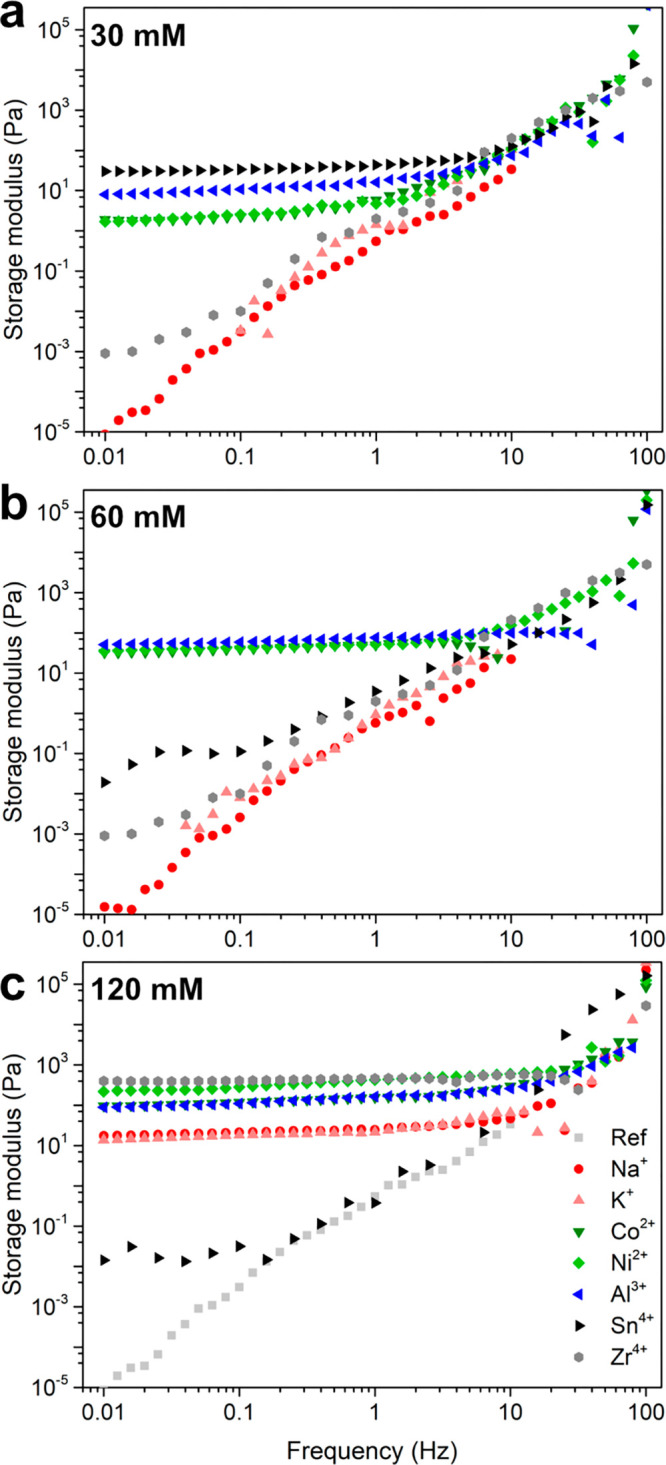
Effect of metal ions with different concentrations (a) 30 mM, (b) 60 mM, and (c) 120 mM on the storage modulus of the WPI solution heated at 90 °C, pH 2 for 24 h. All the PNF/PNF-metal ion solutions were first evaluated after 24 h of incubation in order to ensure that all metal ions hydrolysis had reached equilibrium.

Figure 4 shows the storage modulus of the PNF suspensions/hydrogels formed with, or without, the presence of the metal ions at the concentrations of 30, 60, and 120 mM. The modulus was monitored over the frequency range from 0.01 to 100 Hz. The results were mostly in agreement with the viscous appearance of the samples shown in Figure 1 and displayed a stable storage modulus that could be observed over the entire frequency range due to formation of homogeneous hydrogels (Figure 4). In contrast, the reference solution that had been formed without the presence of metal ions during the growth of the PNFs (shown in Figure 4c as Ref) displayed a constantly increasing storage modulus over the same frequency range.

The rheology data also revealed some specifics about the strength of the hydrogels formed at the different concentrations of the various metal ions. In Figure 4a, it can be seen that the Na^+^ and K^+^ as monovalent metal ions, in the p*K*_a_ range 10–15, did not contribute to any typical hydrogel behavior at 30 mM. The red and the pink symbols follow the light gray symbols of the reference, demonstrating a linear increase in the storage modulus over the frequency range <10 Hz, the region where the viscoelastic response to the applied force is most apparent. The same observation was made at the 60 mM concentration (Figure 4b), whereas for the 120 mM solutions, the trend was broken, and both the Na^+^ and the K^+^ samples displayed a stable storage modulus of *ca*. 20 Pa over the entire frequency range tested (Figure 4c and Table 1). The divalent Co^2+^ and Ni^2+^ ions (p*K*_a_ ≈ 5–10) revealed that the storage modulus of the hydrogels increased from *ca*. 5, to 50, and further to 150–400 Pa for the concentrations of 30, 60, and 120 mM, respectively. The same behavior was observed for Mg^2+^ (not shown). The trivalent Al^3+^ ion in the p*K*_a_ range 1–5 further confirmed the trend with a storage modulus increasing from 15, to 80, and to 150 Pa for the same concentrations. The tetravalent Sn^4+^ metal ions, with the p*K*_a_ values in the lowest range (p*K*_a_ < 1), formed the hydrogel with the highest storage modulus among the samples tested at 30 mM concentration (50 Pa). However, the Sn^4+^ samples at 60 and 120 mM showed unstable storage modulus over the entire frequency range. This was due to the phase separation visible in Figure 1, which made the modulus data interpretation difficult. Likewise, the Al^3+^ sample at 120 mM also showed some phase separation (Figure 1), which decreased the storage modulus value to some degree because of Al^3+^ precipitating as a solid phase. Overall, it could be confirmed that the rheology data describing the viscosity and strength of the hydrogels correlated well with the p*K*_a_ values in Table 1 and the appearances of the hydrogel formation in Figure 1. Occasionally, the hydrogel strength associated with the different metal ions, having similar p*K*_a_ values, scattered along the general trend described above due to different abilities to form metal hydroxides/oxides.

Two metal ions behaved differently in terms of affecting the PNF hydrogel formation described above, that is, iron and zirconium with p*K*_a_ values of 2.2 and −0.3, respectively. No hydrogel formation occurred in the iron-containing samples, regardless of the iron concentration used, and the PNF solutions remained as low-viscous liquids with consistency similar to water and therefore were not included in the rheological evaluation. This was consistent with a limited amount of PNFs in the 30 mM iron sample, or complete absence of PNFs in the other iron containing samples (Figure S5), which suggests that the iron may have interacted with the carboxylic acid groups on the protein chains. These interactions could include the formation of tri/pentanuclear carboxylate complexes with low charge,^52,53^ which have been reported to be stable at low pH values (< 2).^54,55^ The explanation is in agreement with the result that no Fe^2/3+^ ions, in the form of chloride salts, or formed oxides, could be observed in the X-ray diffraction patterns (Figure S1). Zr^4+^ ions required a much greater concentration than expected to form a firm hydrogel (120 mM). Similar to the iron case, Zr^4+^ can interact with water forming highly charged tetranuclear hydrolyzed species,^56^ which may coordinate with carboxylic acid groups, thereby generating carboxylate clusters (which is not the case for Sn^4+^).^57^ However, the highly charged nature of the zirconium ions and the equilibrium with its associated precipitated hydroxides made it difficult to explain the formation of the firm hydrogel at 120 mM (Figure S4), while at lower concentrations, no gel formation occurred. It is noteworthy that some metal ions (Fe^2/3+^ and Zr^4+^) appear to occasionally form intermediate hydroxylated species that interact and inhibit the fibrillation and consequently formation of hydrogels.

The presence of metal ions during the PNF growth and their effects on rheological measurements have previously been addressed only in a few works.^26,42,55,58−65^ Loveday *et al*. suggested that the viscosity of the PNF solutions was associated with the volume fraction of PNFs and the degree of intrafibril entanglements.^26,64^ It was also proposed that Ca^2+^ ions resulted in more significant PNF nucleation and an increase in the amount of short and curved PNFs, thereby allowing for strong fibril networks to form, which contributed to hydrogels demonstrating high viscosity.^26^

### The Gelation Process Kinetics

The acidity of an arbitrary metal cation is determined by its charge to the power of two, radius and electronegativity. The charge has the greatest effect on the derived p*K*_a_ value (eqs 1 and 2), although a smaller radius also affects the p*K*_a_ values due to the charge being distributed over fewer coordinated water molecules (Figure 3a, inner shell), thereby inducing hydrolysis more easily. The coordination and organization of water molecules in the vicinity of the differently sized metal cations were previously described by Baes, Messmer and Wulfsberg *et al*.^43,44^ Omta *et al*.,^47^ Soper *et al*.,^48^ Bylaska *et al*.,^49^ Marcus,^50^ and Mähler *et al*.^51^ suggested that a first hydration shell exist around most ions, even large monovalent ones (*e.g*., Na^+^), whereas well-defined second hydration shells are common around more highly charged ions, for example, Al^3+^ (Figure 3a). However, it is important to underline that when the investigated metal ions were added after the growth of the PNFs, Figure S4), regardless of the PNF morphology (defined as straight or curved fibrils),^31^ no gelation occurred in any of the samples (Figure S4). This demonstrates that the effects of the metal ions during the growth stage of the PNFs are of central relevance, and the PNF fibrillation kinetics was therefore further studied.

**Figure 5:**
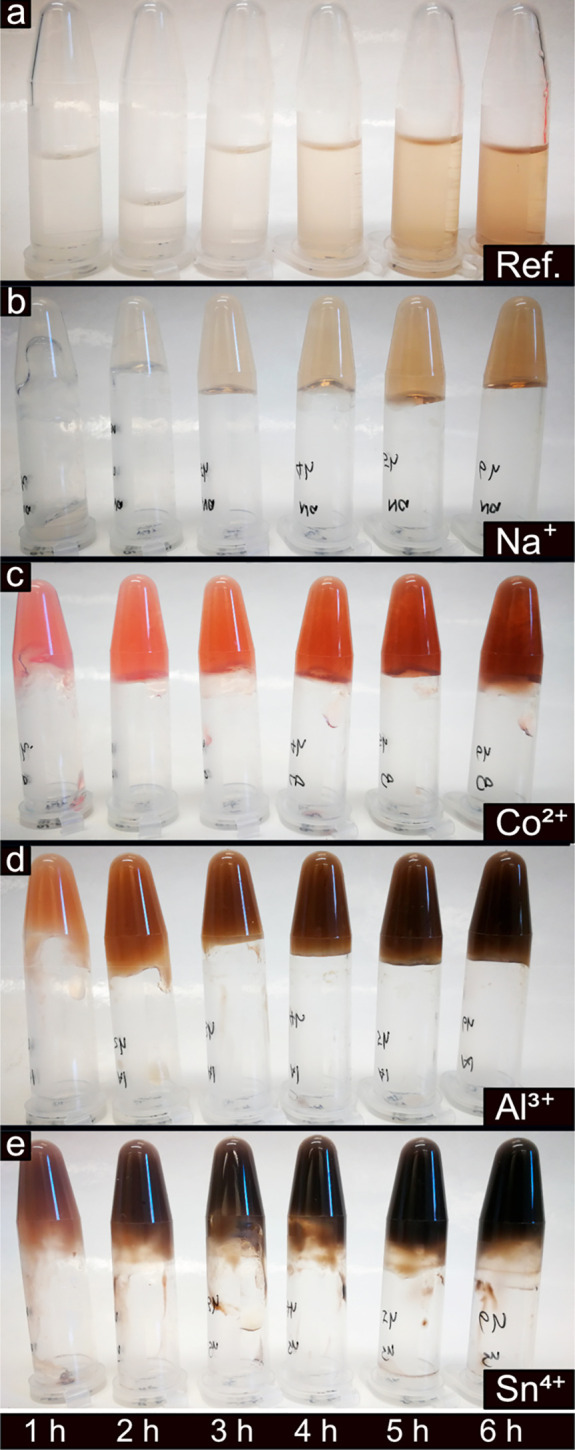
WPIs after incubation at 90 °C of for 1–6 h with and without the presence of different metal ions at 120 mM concentration.

Figure 5 shows the gradual gel formation (with time) for the samples with and without the presence of metal ions during the first 6 h of the PNF growth. The starting solutions were adjusted to pH 2 before the additions of the metal ions. All the metal-ion-containing samples (except Na^+^) formed hydrogels within 40 min, while the Na^+^ sample required around 1 h (Figure 5). The color changes with the fibrillation time for the Na^+^ sample followed the reference (Figure 5a,b), indicating that the presence of Na^+^ ions had a limited effect on the PNF growth other than structurally organizing the water molecules (Figure 3a). For more charged metal ions (Co^2+^ and Al^3+^) at the same concentration (120 mM), a gradual darkening of the samples was observed compared to the Na^+^ sample, see Figure 5c,d. More noticeably, the Sn^4+^ sample turned black after 6 h of PNF growth (Figure 5e). This extensive darkening was presumed to occur due to acidic PNF degradation and oxidation.

**Figure 6:**
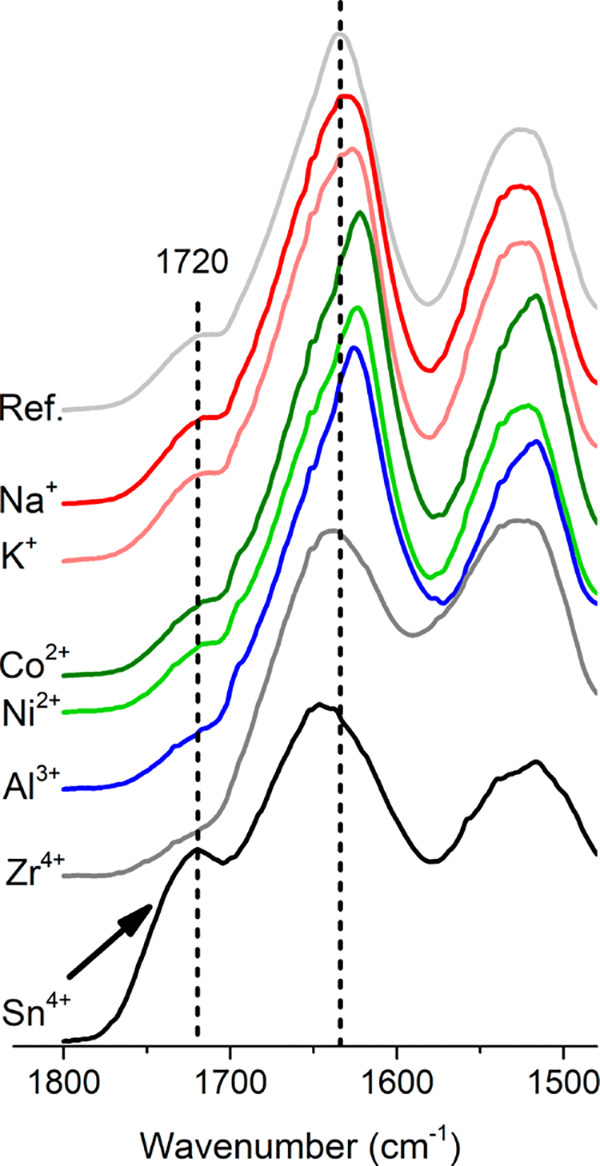
Protein structures of the samples with and without metal ions (120 mM) after 24 h incubation. Samples were freeze-dried and stored in a desiccator before the measurement. IR spectra were normalized according to the amide I peak.

**Figure 7:**
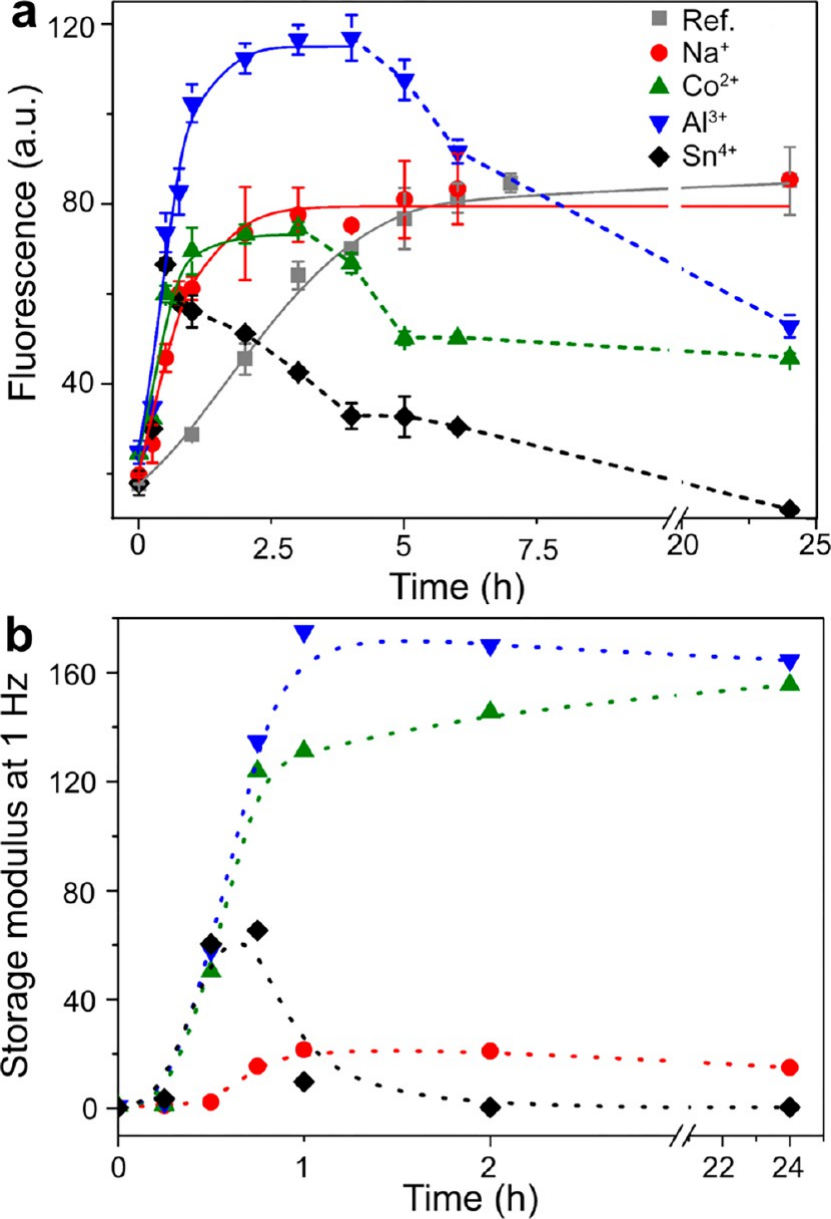
(a) Effect of metal ions (Na^+^, Co^2+^, Al^3+^, and Sn^4+^ at 120 mM) on the kinetics of WPI fibril formation at pH 2, 90 °C, measured by ThT fluorescence at 485 nm. (b) Gelation data for WPI solutions with metal ions at 120 mM.

Overall, the addition of metal ions with lower p*K*_a_ values resulted in more acidic conditions, always contributing to even darker solutions. This darker appearance of the fibril solutions with more acidic conditions was previously ascribed to degradation in the case of polysaccharides, for example, cellulose fibrils.^66^ Infrared spectroscopy (IR) was therefore used to investigate the possible extensive formation of carboxylic acid groups associated with the oxidation/hydrolysis of the PNFs, shown as a carbonyl group peak at 1720 cm^–1^, see Figure 6.^67,68^ The peak was most visible for the PNFs grown in the presence of the Sn^4+^ ions at a concentration of 120 mM, that is, the most acidic condition (pH = 0.7) among all the samples (Figure 6 and Table 1). At this pH, extensive protein hydrolysis has been previously reported, resulting in significant amounts of carboxylic acid groups.^69^ The carbonyl groups were however not evident in the case of the Zr^4+^, suggesting that extensive degradation had not occurred in this sample and was then only dominant in the Sn^4+^ case, see Figure 6. It is also shown in Figure 7b that the Sn^4+^ sample lost its storage modulus after *ca*. 1 h of incubation.

Figure 6 also shows that the protein amide I peak at *ca*. 1640 cm^–1^ shifted to lower/higher wavenumbers compared to the reference peak position indicated by a dashed line. These shifts suggest changes in the protein secondary structures.^70,71^ A shift toward lower wavenumbers indicates an increase in the formation of β-sheet features in the PNFs, thus resulting in a more organized protein structure.^70,71^ This was observed as associated with the mono-, di-, and trivalent metal ions (K^+^, Na^+^, Co^2+^, Ni^2+^, Al^3+^), and the shift was most pronounced in the case of divalent ions, compared to the mono- and trivalent ions (ref < Na^+^ < K^+^ < Co^2+^, Ni^2+^ > Al^3+^). The addition of tetravalent metal ions resulted, however, in more unordered/random coils structures, as suggested by the shifts occurring toward higher wavenumbers. These results indicate strong interactions between metal ions and the protein during the formation of PNFs, thereby impairing the formation of more organized structures within the protein itself.

ThT fluorescence was used to monitor the amount of PNFs formed during the incubation and study the fibrillation kinetic parameters in the systems with, and without, the presence of metal ions at a concentration of 120 mM (Figure 7a). The fluorescence of the reference sample increased continuously during the first 5-7 h, reaching a plateau at *ca*. 85 fluorescence units (FU) at 7.5 h, see Figure 7a. The Na^+^ sample reached a similar plateau in a significantly shorter time: 3.5 h, that is, half of the time needed to reach the same plateau as for the reference sample. For the Co^2+^ and Al^3+^ samples, most of the PNF growth occurred during the first 2 h, reaching *ca*. 75 and 115 FU as maximal values, respectively. These samples also showed a decrease in the fluorescence after 3 h of incubation time to *ca*. 50 FU (Figure 7a). The Sn^4+^ sample initially reached a similar maximal fluorescence intensity as the Co^2+^ sample, but a decrease in the fluorescence occurred with time from the maximal value after 30 min (*ca*. 74 FU) until reaching *ca*. 20% of its maximum value at 24 h (Figure 7a). The decrease in ThT fluorescence signal after long incubation time was previously observed for the formation of PNFs from potato and β-lactoglobulin proteins.^39,65^ It was then attributed to a local gelation/precipitation of the PNFs and/or the breakdown of the fibril structure.^65^ In the presence of the metal ions, it was found that the relative decrease in the signal strength increased with decreasing p*K*_a_ of the metal ions, in the order Co^2+^, Al^3+^, and Sn^4+^ (Figure 7a). The more limited fluorescence with time was therefore interpreted as a result of an extensive PNF coagulation occurring with the simultaneous precipitation of the metal hydroxides and oxides, together with protein hydrolysis due to the high acidity of the Sn^4+^ system that may have hindered further PNF fibrillation. This explanation agrees with the values of the storage modulus at 1.0 Hz shown in Figure 7b, where the Sn^4+^ sample lost its gel-like properties as the sample was incubated for *ca*. 0.5–1 h. A small drop in the storage modulus was also observed for the aluminum sample, which also displayed a separation of solid phase after the first hour of incubation (see Figures 1 and 7b). However, this decrease in storage modulus was small, and the sample mostly displayed a rheological behavior in agreement with the 120 mM Co^2+^ and Na^+^ samples, which maintained gel-like properties over the entire 24 h incubation measurement. The results suggest that the decrease of ThT fluorescence acts as an early indication of hydroxide/oxide phase separation on a finer nanometre resolution level, while the rheological and diffraction properties more reflect the nature of the samples on a macroscopic level.

The PNF-Sn^4+^ sample data were not possible to fit with the Finke–Watzky model, and therefore the kinetic parameters were calculated using linear regression with the data obtained at 0.25 and 0.5 h. The calculated parameters from the fitting, that is, *t*_1/2_, , and *t*_lag_, are shown in Table 2. In general, the PNF always formed in significantly shorter times when metal ions were present, as compared to the reference sample. The results for the reference sample show that 2.3 h was needed to grow 50% of the total amount of PNFs (*t*_1/2_, see Table 2). The use of 120 mM Na^+^, with the highest p*K*_a_ among the tested metal ions (*ca*. 14), resulted in a *t*_1/2_ equal to 0.62 h. A decrease in the p*K*_a_ values for the Co^2+^ and Al^3+^-containing samples (as compared to the Na^+^) reduced even further the time needed to grow 50% of the PNFs, being 0.44 and 0.55 h, respectively. The lowest *t*_1/2_ value among all the samples was found for the Sn^4+^ ion, showing a *t*_1/2_ value of 0.33 h. Accordingly, the Sn^4+^ ions were able to promote the growing of half of the maximum amount of the formed fibrils in 14% of the *t*_1/2_ obtained for the reference sample. The trend was verified from the maximum rate of fluorescence (), which increased with the decrease of the p*K*_a_ values of the metal ions (Tables 1 and 2). The PNF nucleation time in Table 2, that is, time to reach the detectable amount of PNFs (*t*_lag_), revealed that all samples containing metal ions nucleated faster than the reference sample. The increase in the *t*_lag_ when metal ions with low p*K*_a_ values are used, for example, from 0.002 (Na^+^) to 0.168 (Sn^4+^), is suggested as a consequence of the larger hydration volume and/or a readily formation of metal hydroxides/oxides for the most acidic metal ions (*e.g*. Sn^4+^), which may delay their initial interaction with the protein functional groups during the PNF growth.

**Table 2:** Kinetic Parameters Obtained by Fitting eq 1 to ThT Fluorescence Data: *t*_1/2_, , and *t*_lag_

samples	*t*_1/2_ (h)	(FU h^–1^)	*t*_lag_ (h)	adjusted *R*^2^
ref	2.34	16.2	0.26	0.995
Na^+^	0.62	50.9	0.002	0.975
Co^2+^	0.44	62.1	0.043	0.929
Al^3+^	0.55	97.6	0.085	0.977
Sn^4+^	0.33	146.6	0.168	0.999

**Figure 8:**
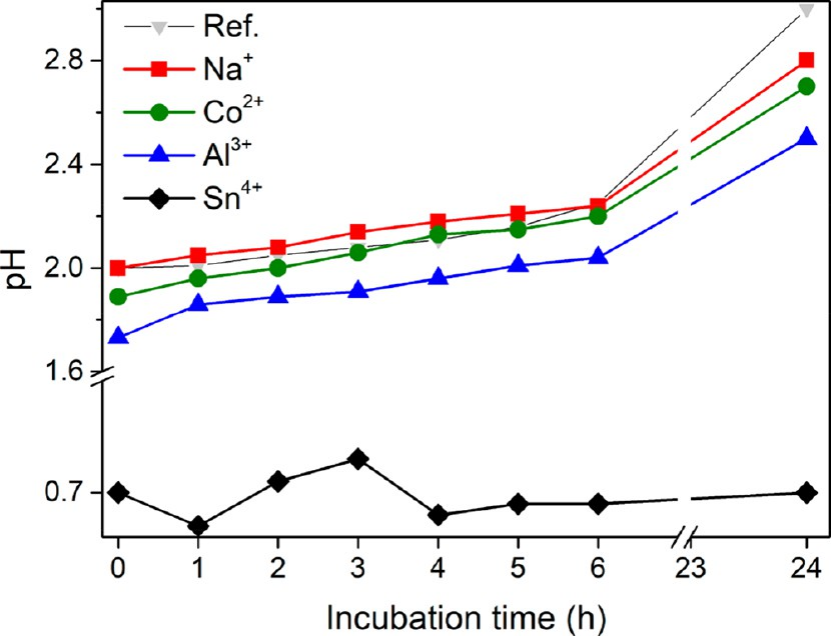
pH of WPI solutions after incubation for 1–24 h with and without the presence of 120 mM Na^+^, Co^2+^, Al^3+^, and Sn^4+^.

Figure 8 shows the acidity of the PNF solutions in Figure 5 over the first 6 h of incubation. The immediate effect of the addition of the metal ions was a drop in pH, which became more significant with a decreasing p*K*_a_ value of the metal ions (see Figure 8, 0 h). The lowest pH value was observed for the Sn^4+^-PNF sample (pH = 0.7), which was due to the complete Sn^4+^ hydrolysis, followed by the formation of SnO_2_ (see X-ray diffraction in Figure S1d). The pH over the incubation time increased continuously for most of the metal ions, similar to the reference PNF solution. This increase in pH over the incubation time is due to the formation of PNFs and the hydrolysis of the peptide bonds in the proteins, see Figure 3d. The pH for the Sn^4+^ metal ion-PNF solution remained relatively constant over the entire incubation time, that is, at pH ≈ 0.7. This was due to the extensive formation of hydroxides and oxides with the release of hydronium ions (H_3_O^+^, Figure 3), which was more dominant in overlapping the increase in the pH because of the hydrolysis. This trend showed most explicitly for the measurements taken over 6 h (Figure 8).

A fast fibrillation was previously observed by Ikeda *et al*.^59^ and Veerman *et al*.^60^ and was suggested as a result of a reduced electrostatic repulsion between the growing PNFs, together with an enhanced fibril nucleation.^61,72^ The significance of reducing the electrostatic repulsion was herein confirmed and displayed as low ζ potential values at the initial stage of the protein fibrillation for samples containing the metal ions, see Figure S3. The accelerated PNF growth also seemed to be related to the ability of the metal ions to generate hydronium ions (H_3_O^+^), which in turn favored the protein hydrolysis (Figure 3d) and further the PNF formation and growth.^42,62^

### Protein Nanofibril Morphology

**Figure 9:**
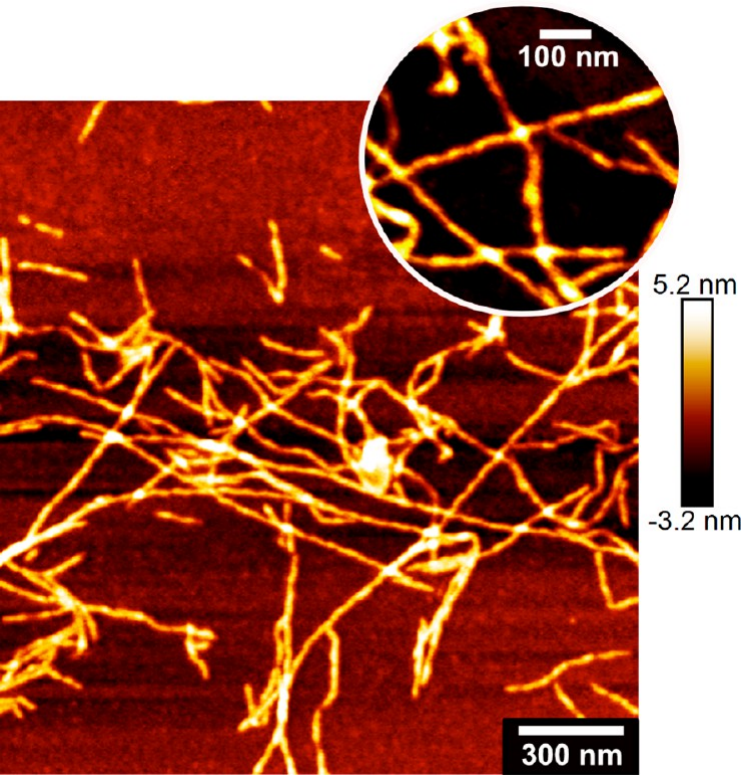
AFM image of WPI fibrils grown at pH 2, 90 °C, after 24 h.

**Figure 10:**
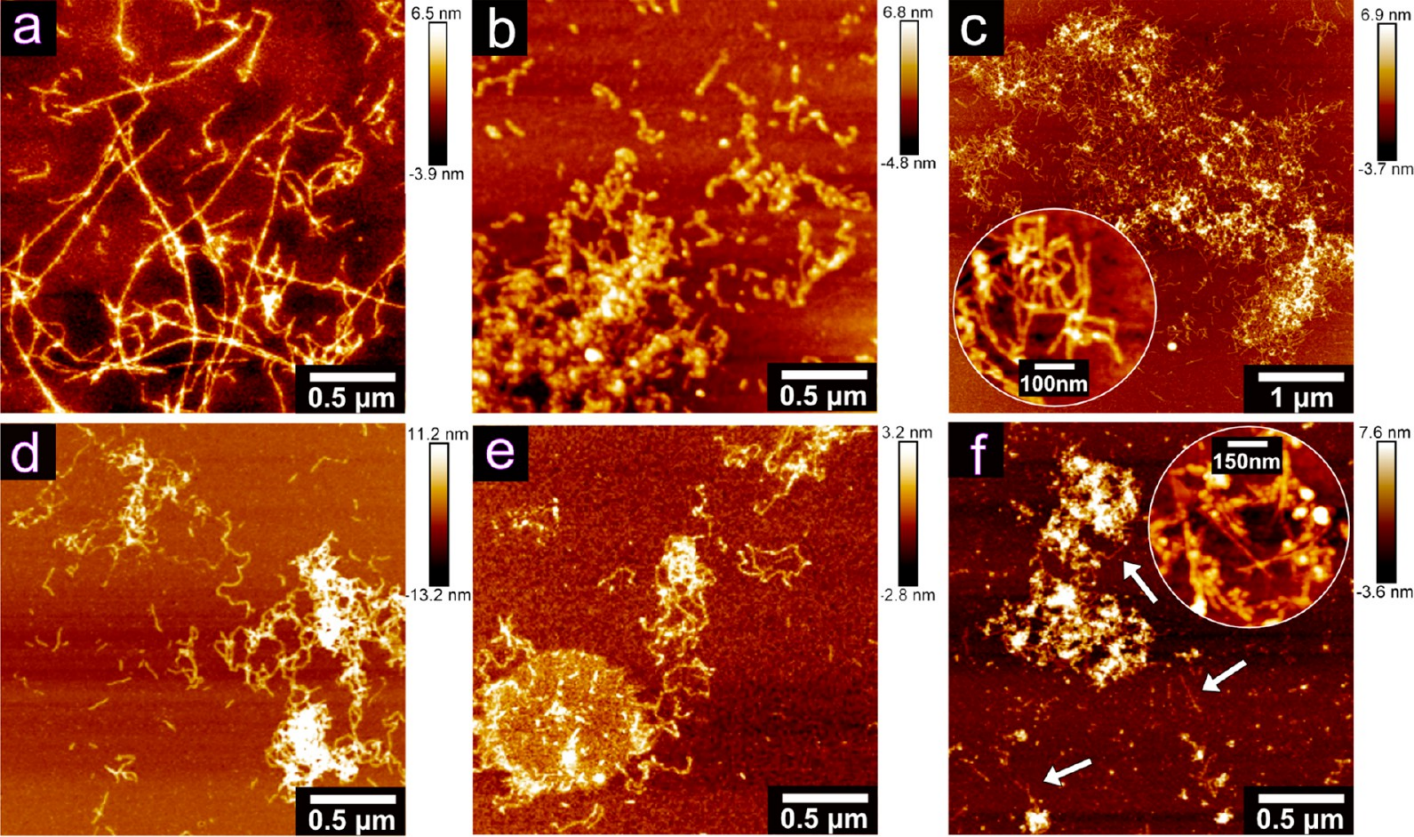
AFM images of whey protein PNFs grown with and without the addition of different metal ions under pH 2, 90 °C of for 24 h. PNFs grown with (a) 30 mM Na^+^, (b) 60 mM Na^+^, (c) 120 mM Na^+^, (d) 30 mM Ni^2+^, (e) 30 mM Al^3+^, and (f) 30 mM Sn^4+^.

Figure 9 shows the morphology of the PNFs grown in the absence of metal ions. The PNFs varied between *ca*. 200 nm and 4 μm in length, while being dominantly long and straight fibrils with limited entanglements and aggregation. The thicknesses of the individual fibrils were estimated to *ca*. 4–5 nm, which is consistent with previous reports showing fibrils from β-lactoglobulin,^73^ egg white lysozyme,^74^ and soy protein,^36^ showing thicknesses in the range 2–10 nm. The insert in Figure 9 highlights that twisted ribbon fibril morphology could also be observed for the whey protein in this work. This morphology was similar to that previously described for lysozyme and β-lactoglobulin by Mezzenga *et al*.^75^ and Guzzi *et al*.^55^ The PNFs, grown in the presence of 30 mM Na^+^ and K^+^ ions, formed as relatively straight and long fibrils (Figures 10a and S5), resembling the reference PNFs. An increase in the ion concentration to 60 mM or 120 mM resulted in shorter and curved PNFs for the same metal ion, suggesting that a concentration dependence also existed, see Figure 10b,c. Curved fibrils formed in the presence of Na^+^ ions were previously observed by Loveday *et al*.^26,42^ As the concentration of the monovalent ions increased, the tendency of the fibrils to aggregate also increased. The stronger tendency to aggregate partly explained the increase in the viscosity of the solutions (Figures 1 and 3), that is, favoring the formation of PNFs entangled as a network.^76^

Figure 10c shows the size of a typical aggregate (*ca*. 5 μm in width), while the inset displays in more detail the fibrils as entangled entities. Figure 10d shows that the curved fibrils were formed already at a concentration of 30 mM when divalent metal ions (Ni^2+^, Co^2+^*etc*.) were present during the growth of the PNFs. Similarly, for trivalent Al^3+^, much smaller and curved fibrils were formed, see Figure 10e, as compared to the reference PNFs. For the tetravalent 30 mM Sn^4+^ sample, the fibrils appeared shorter (*ca*. 250 nm) than those in the Ni^2+^, Co^2+^ (Figure S5), and Al^3+^ samples (at the same concentration), which all showed PNFs ranging up to *ca*. 500 nm in length. The curved and also entangled PNFs in the case of polyvalent metal ions made the determination of the accurate PNF length difficult. Overall, the trend was decreasing fibril length with increasing charge and smaller radius of the metal ion present during the PNF growth (*i*.*e*., lower p*K*_a_ value), see Figure S6. This could be due to the faster protein hydrolysis, promoted by the solvation of the metal ions, which resulted in more nucleation and in turn shorter PNFs. The arrows in Figure 10f highlight the presence of smaller, short, curved, and entangled PNFs in the Sn^4+^ ion sample. Finally, large and condensed networks, as shown in Figure 10c, were always observed in all samples with polyvalent metal ions.

### Release of Metal Ions from the PNF Hydrogels

**Figure 11:**
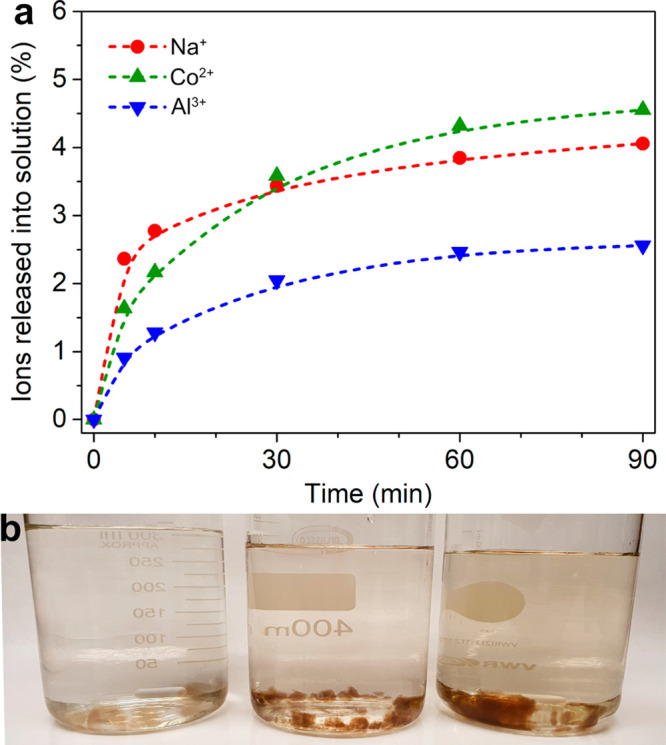
(a) The kinetic study of the release of Na^+^, Co^2+^, and Al^3+^ from PNF hydrogel in Milli-Q water. (b) The PNF hydrogels containing Na^+^ (left)/Co^2+^ (middle)/Al^3+^ (right) immersed in Milli-Q water for 90 min.

Figure 11 shows the rate of the release of Na^+^, Co^2+^, and Al^3+^ ions, illustrating the binding strength and/or locking capacity of the metal ions in their different structural configurations with the PNFs in the hydrogels (over 90 min). The release rate of the Na^+^, Co^2+^, and Al^3+^ ions showed a similar trend (Figure 11a), that is, the amount of ions released into Milli-Q water increased rapidly during the first 10 min and leveled off after 30 min. The releasing rate of the metal ions decreased with the increase of the p*K*_a_ value of the metal ion, that is, Na^+^ > Co^2+^ > Al^3+^ during the first 10 min. At the end of the measurement (after 90 min), around 4% of Na^+^ and Co^2+^ ions had been released from the PNF hydrogel, while only 2% of Al^3+^ ions were detected as released from the hydrogel in the Milli-Q water, see Figure 11a. It is therefore suggested that the lower charged metal ions with greater p*K*_a_ values were less strongly associated with PNFs in the hydrogels, possibly being dominated by outer-sphere hydration interactions with the PNFs (Figure S7). It is also noteworthy that the majority of the ions, that is, more than 96%, remained as associated with the PNF hydrogels, explaining why the hydrogels kept their initial color and shape (remaining as a hydrogel, Figure S8) after 90 min of immersion in Milli-Q water and constant stirring, see Figure 11b. In the second set of samples, the same test was carried out for the hydrogels as when immersed in the Milli-Q water. The only difference was that the water had been acidified to pH 2, demonstrating identical trends of metal ions release (and hydrogels state) as for the Milli-Q water (above), with the difference that each sample released 10 times more ions (Figure S9).

**Figure 12:**
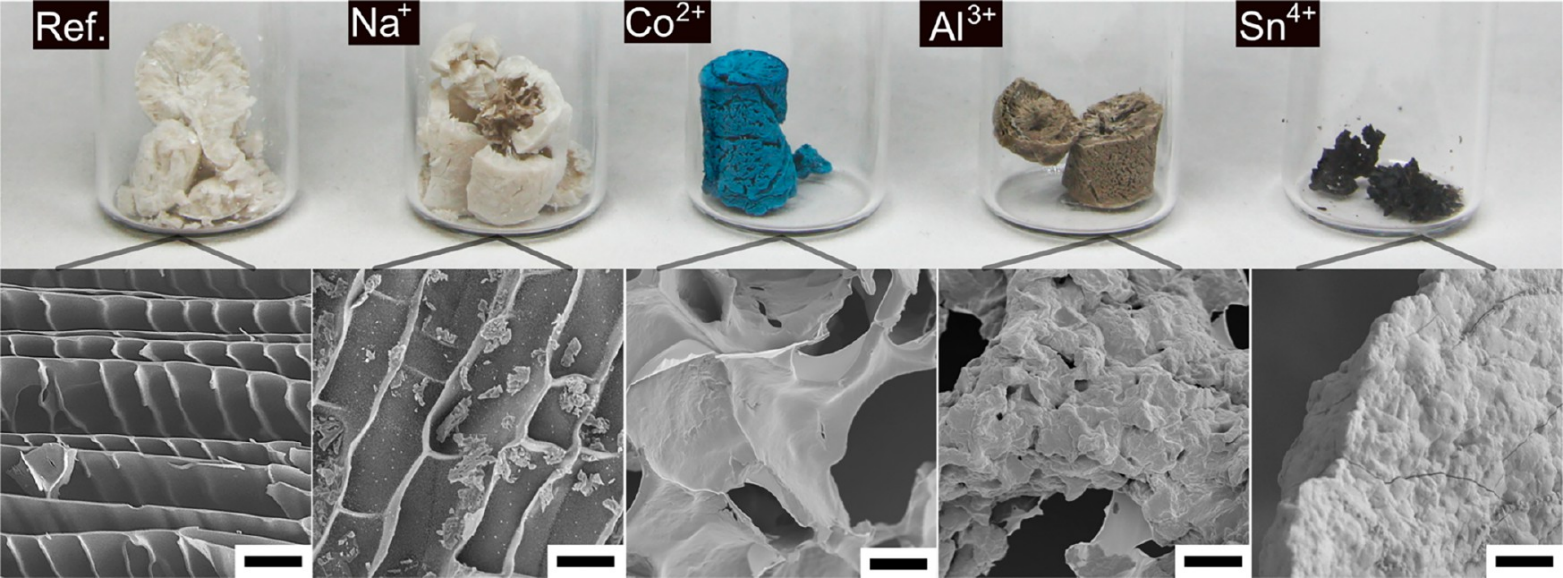
The freeze-dried PNF samples (1 mL) with and without the presence of 120 mM metal ions (Na^+^, Co^2+^, Al^3+,^ and Sn^4+^). The inserted scanning electron microscopy (SEM) images showed a detailed structure of the foams. The scale bar is 10 μm.

Figure 12 shows the appearance of the lyophilized PNF hydrogels prepared with and without the presence of the metal ions. All the gels, except the gel grown in the presence of the Sn^4+^ ions, developed into structural foams with 72 h of freeze-drying. The reference and the Na^+^ foams displayed a similar whitish color and a brittle behavior, and these foams mostly broke during the transfer from the Eppendorf tubes to the glass beakers, shown in Figure 12.^46,77^ On the contrary, the Co^2+^ and Al^3+^ foams maintained to a large extent the shape of the Eppendorf tube, although a volumetric shrinkage of *ca*. 20% was observed as compared to the reference sample. For these samples, a very bright blue and silver-gray color indicated a uniform distribution of the metal ions, respectively, penetrating the entire samples. The shrinkage of the hydrogels with freeze-drying became more evident for the hydrogels grown in the presence of metal ions with lower p*K*_a_ values. The foam produced from the lyophilization of the hydrogels with Sn^4+^ ions resulted in the most significant shrinkage (*ca*. 80%) among the aforementioned. This sample was fragmented into several hard and black pieces, which reflected the short and curved fibril morphology associated with possible degradation resulting from the addition of the Sn^4+^ ions (see Figures 5, 6, and 10). The foam morphologies of the lyophilized reference foam and the Na^+^ sample suggest the formation of elongated ice crystals during the rapid freezing (in liquid nitrogen) of the hydrogel, which resulted in PNF cell walls the size of *ca*. 0.2 μm (Figure 12). The microstructures of the Co^2+^ and Al^3+^ foams show that the ice crystals grew more isotropically in these samples, compared to the reference and Na^+^ samples. The cell wall of the Al^3+^ foams was thicker (thickness *ca*. 2 μm) than all samples mentioned before (*i*.*e*., reference Na^+^ and Co^2+^), featuring a much rough surface (Figure 12). The micrograph from the Sn^4+^ sample also reveals a course structure with a rougher surface and no pores observed, corresponding to a collapsed structure and demonstrating the clear phase separation and/or embedding of the PNFs taking place in these samples. The lyophilized 30 and 60 mM Al^3+^ and Sn^4+^ PNF foams showed a smoother surface (Figure S10) compared to the foams from the 120 mM samples, suggesting that the PNF foam morphology have been affected by the formation of aluminum hydroxide (Figure S2) and SnO_2_ (Figure S1).

## Conclusions

The correlation of the viscosity observations, the rheology data results (storage modulus), acidity changes with different metal ion p*K*_a_ values (pH variation), and the subsequent differences in the formation of PNF hydrogels was demonstrated. First and foremost, it was shown that the addition of the metal ions before the PNF growth is a key factor for the coordination of the growing PNFs with the hydrolyzed metal ions and the water molecules, resulting in different gelation behaviors related to the different nature of the metal ions. Namely, none of the evaluated metal ions were capable of forming hydrogels by themselves at the concentrations evaluated, or on the addition of the same ions at the same concentration after growth of the PNFs (Figure S4). It was also demonstrated that the strength of PNF hydrogels was strongly dependent on the concentration and p*K*_a_ values of the metal ions present during the PNF growth. Monovalent Na^+^ and K^+^ ions (p*K*_a_ ∼14) only showed mild effects on the viscosity of the PNF solutions as compared to divalent Ni^2+^, Mg^2+^, and Co^2+^ ions (p*K*_a_ 5–11) that made the PNF solutions to develop into strong hydrogels. This trend prevailed as long as the acidity of the metal ions did not result in excessive precipitation of solid-phase metal hydroxide/oxides. The behavior was observed over the p*K*_a_ range from the most acidic Sn^4+^ ion (p*K*_a_ −0.6) to the nonacidic K^+^ ion (p*K*_a_ 14). The higher storage modulus associated with metal ions with lower p*K*_a_ values indicated a stronger structural network of PNFs formed during the gelation, which was supported by a slower release of Al^3+^ compared to that of Na^+^ and Co^2+^ (Figure 11), and the formation of more dense foams of Al^3+^-PNF after lyophilization (Figure 12). The rate of the fibril formation increased almost 10 times with the decreasing p*K*_a_ values compared to the reference sample (Table 2). The faster growth of the PNFs was reflected in the morphology of the fibrils, which developed into much smaller and curved versions (*ca*. 0.5 μm in length), from being long and straight fibrils (*ca*. 5 μm in length) when grown in absence of metal ions. With consideration to the results showing increasing hydrogel strength with lower p*K*_a_ values and limited structural foam properties (after lyophilization, see Figure 12) for the most charged and smallest metal ions (lowest p*K*_a_ values), it is suggested that the hydrogel properties were in fact related mostly to the coordination of metal ions to the surface of the PNFs rather than to the existence solely of entangled PNF networks.

## Methods

### Materials

Whey protein isolate (WPI) (Lacprodan DI-9224) was provided by Arla Food Ingredients. Hydrochloric acid (HCl) and the chloride salts (*i*.*e*., NaCl, KCl, CoCl_2_, NiCl_2_, AlCl_3_, FeCl_3_, SnCl_4_, and ZrCl_4_), with a purity above 99%, were all purchased from Sigma-Aldrich. The dialysis membranes (cutoff of 6–8 kDa and 100 kDa) were purchased from Spectrum Laboratories, Rancho Dominguez, CA.

### Preparation of PNF Hydrogels

WPI was dissolved in 0.1 M HCl under constant magnetic stirring, with a concentration of 100 g/L. The solution was dialyzed against 0.01 M HCl (pH 2), using a membrane with a molecular weight cutoff of 6–8 kDa, for 24 h at room temperature to remove residual salts. The dialyzed solution was then diluted to 40 g/L and adjusted to pH 2 followed by incubation at 90 °C for 24 h. For the preparation of the PNF in the presence of the metal ions, the different chloride salts with selected concentrations (*i*.*e*., 30, 60, or 120 mM) were added before incubation at 90 °C for 24 h. The maximum salt concentration was limited to 120 mM to avoid hydrogel phase separation due to the precipitation of the salts.^63^

### Rheology

The rotational test was performed at 25 °C using a DHR-2 rheometer (TA Instruments, USA), fitted with 25 mm diameter stainless steel parallel plates. After heating for the required time, an aliquot of the sample was taken from the oven and placed in an ice water bath for at least 10 min before the test. The sample was then transferred to the rheometer base plate, and a preshear step was performed at a shear rate of 200 s^–1^ for 120 s to erase the sample’s shear history. Samples were allowed to reach equilibrium for 1 min before being subjected to an amplitude sweep (0.01–100% strain at a frequency of 1 Hz) and a frequency sweep (0.01–100 Hz). The strain value used in frequency sweep was chosen from the linear region of the amplitude sweep.

### Transmission Electron Microscopy

A droplet (3 μL) of a diluted WPI nanofibril suspension was placed on a Formvar/carbon-coated copper grid (200 mesh Formvar-carbon, Ted Pella, USA) for ca. 5 min before removing the droplet with a filter paper. The sample was then allowed to dry under ambient conditions and examined with a Hitachi HT-7700 high-resolution microscope operated at 100 kV.

### Thioflavin T Fluorescence

For the fluorescence measurements, 0.2 mL of the samples were well stirred before mixing with 2.4 mL of a 50 μM thioflavin T (ThT) solution, which were carried out with a Cary Eclipse Spectro fluorometer (Varian) using excitation at 440 nm and recroding the emission spectra between 460 and 600 nm. The experiments were repeated for 2–4 times for each sample. The time-dependent ThT fluorescence data, after subtraction of the fluorescence intensity at *t* = 0, was fitted to the Finke–Watzky eq 3:^78^
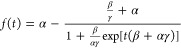
3where *f*(*t*) is the fluorescence intensity at time *t* in hours, and α, β, and γ are arbitrary constants. These constants can be used to calculate the lag time (*t*_lag_), i.e. the time to reach detectable amount of PNFs (eq 4), maximum rate of increase  (eq 5), and the time for the fluorescence intensity to increase to half of its maximum (*t*_1/2_, eq 6) accordingly.
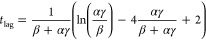
4
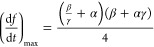
5
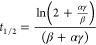
6

### Fourier Transform Infrared Spectroscopy

The samples were freeze-dried for 24 h, before the Fourier transform infrared spectroscopy (FTIR) measurements were made using a PerkinElmer Spotlight 400 FTIR equipped with a Golden Gate (Specac Ltd.) single reflection ATR crystal. The spectra were recorded between 4000 and 750 cm^–1^ with 8 scans and a resolution of 4 cm^–1^.

### Atomic Force Microscopy

PNF samples with or without metal ions were diluted in 10 mM HCl (1:1000), applied onto freshly cleaved mica surfaces and allowed to dry in air. These samples were then investigated by an atomic force microscope (Bruker Corp., USA) in the Scanasyst-air mode, and tapping mode. The images were analyzed using Nanoscope 1.5 software (Bruker).

### Release of Metal Ions from the PNF Hydrogels

Three mL of PNF hydrogels were prepared as described previously in the presence of Na^+^, Co^2+^, and Al^3+^ metal ions at a concentration of 90 mM. The 90 mM concentration was selected as this concentration showed to be suitable for the formation of a stable hydrogel. After incubation for 24 h, the hydrogels were placed in a beaker containing 300 mL of Milli-Q water under continuous magnetic stirring to evaluate the release of ions from the PNF hydrogel. Two mL aliquots were taken from the solution containing the PNF hydrogel after 0, 5, 10, 30, 60, and 90 min, post the initial hydrogel immersion. The aliquots were centrifuged at 12,000 × *g* for 30 min to remove possible hydrogel fragments suspended in the solution. One mL of the supernatant was diluted to 10 mL with 0.01 M HCl solution using a volumetric flask for the inductively coupled plasma measurements. Inductively coupled plasma-optional emission spectrometry (ICP-OES, Thermo Fisher iCAP 7400, USA) was used to measure the concentration of the metal ions in the solution.

### Scanning Electron Microscopy

The PNF hydrogels formed with the presence of different metal ions were frozen in liquid nitrogen for 10 min before lyophilization for at least 48 h. The obtained PNF foam was fixed on an aluminum sample holder and then coated with platinum/palladium for 30 s before examination using a S-4800 field-emission scanning electron microscope (Hitachi, Japan).

## Supporting Information Available

X-ray spectra of all samples; TEM image of aluminum hydroxide; ζ potential data of PNF-metal ion solutions over time; PNF solutions after 24 h fibrillation followed by an addition of 120 mM different metal ions; AFM images of PNFs generated with the presence of different metal ions; end-to-end length distribution of the PNFs formed in the reference, Na^+^, Ni^2+^, Al^3+^, and Sn^4+^ (30 mM) systems; suggested mechanism for the interaction between PNFs, metal ions, and water in the hydrogels; the image of PNF hydrogels immersed in Milli-Q water at the beginning of the release experiment; the kinetic study of Na^+^, Co^2+^, and Al^3+^ ions released from PNF hydrogels in 0.01 M HCl; SEM images of the lyophilized PNF foams with 30 and 60 mM Al^3+^ and Sn^4+^ (PDF)
