# Supplementary material for: Protein Nanofibrils and Their Hydrogel Formation with Metal Ions
Source: ACS Nano. 2021 Mar 5;15(3):5341–54. doi: 10.1021/acsnano.0c10893 (PMC8041371; doi:10.1021/acsnano.0c10893)
Supplement: Supplementary file 1 — nn0c10893_si_001.pdf [file nn0c10893_si_001.pdf]

Supplementary Information for

Protein Nanofibrils and Their Hydrogel Formation with Metal Ions

Xinchen Ye[†]*, Antonio J. Capezza*[†]*, Xiong Xiao*[†]*, Christofer Lendel*[‡]*, Mikael S. Hedenqvist*[†]*, Vadim G. Kessler*[ꝛ] *and Richard T. Olsson*[†]***

[†]Department of Fibre and Polymer Technology, School of Engineering Sciences in Chemistry, Biotechnology and Health, KTH Royal Institute of Technology, SE-100 44 Stockholm, Sweden,

[‡]Department of Chemistry, School of Engineering Sciences in Chemistry, Biotechnology and Health, KTH Royal Institute of Technology, Stockholm SE-100 44, Sweden

[ꝛ]Department of Molecular Sciences, Swedish University of Agricultural Sciences, Box 7015, 750 07 Uppsala, Sweden

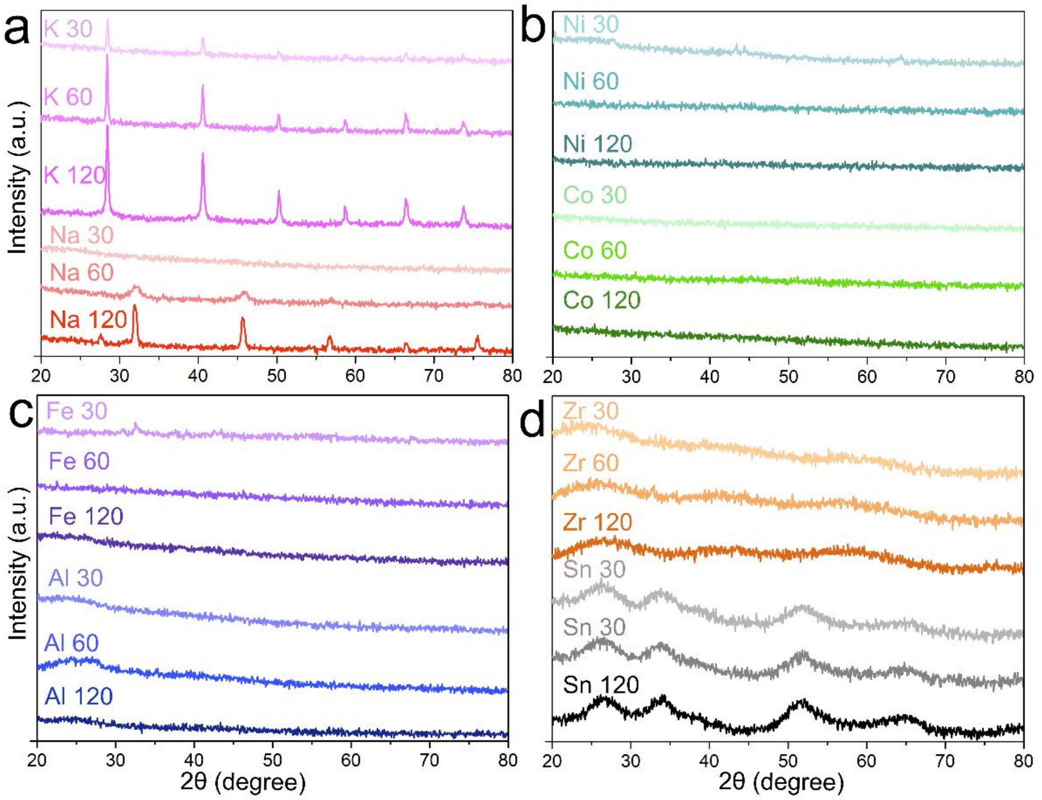

Figure S1. X-ray spectra of freeze-dried PNFs grown with the present of K^+, Na^+ (a); Ni^{2+}, Co^{2+} (b); $Fe^{2/3+}$, Al^{3+} (c) and Zr^{4+}, Sn^{4+} (d). The X-ray diffraction pattern in (a) and (d) indicates the existence of KCl, NaCl and SnO_2, respectively. The full width at half of the maximum intensity (FWHM) of the peak at *ca.* 50 degrees is around 8, 6 and 5 degrees for Sn^{4+} 30, 60 and 120 samples, respectively. These spectra were obtained using an ARL X'TRA X-ray diffractometer with Cu Kα radiation (wavelength: 1.54 Å) at 44 mA and 45 kV.

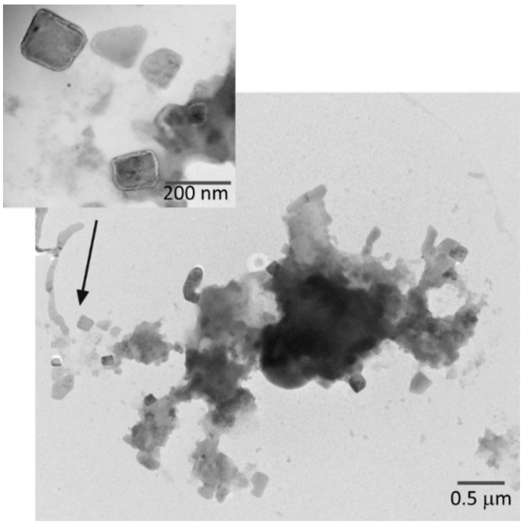

Figure S2. TEM images of aluminium hydroxide.

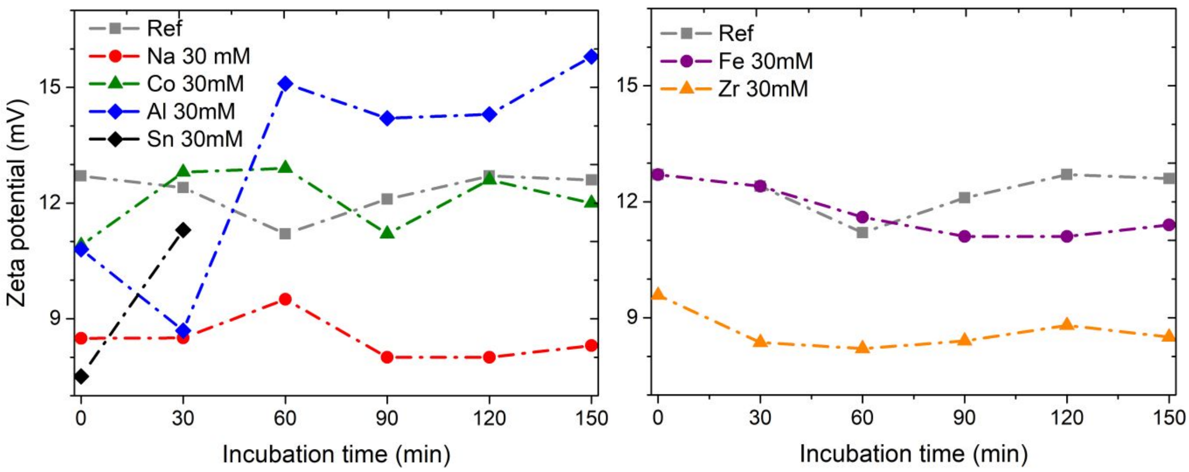

Figure S3. Zeta potential of PNF-metal ion solutions over time.

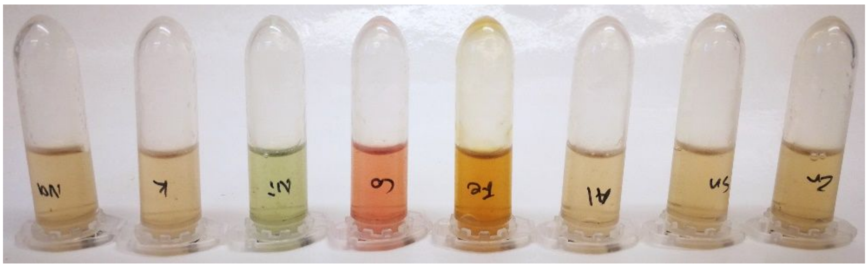

Figure S4. PNF solutions after 24-hour fibrillation followed by an addition of 120 mM different metal ions, *i.e.* Na^{+}, K^{+}, Ni^{2+}, Co^{2+}, Fe^{3+}, Al^{3+}, Sn^{4+} and Zr^{4+} from left to right.

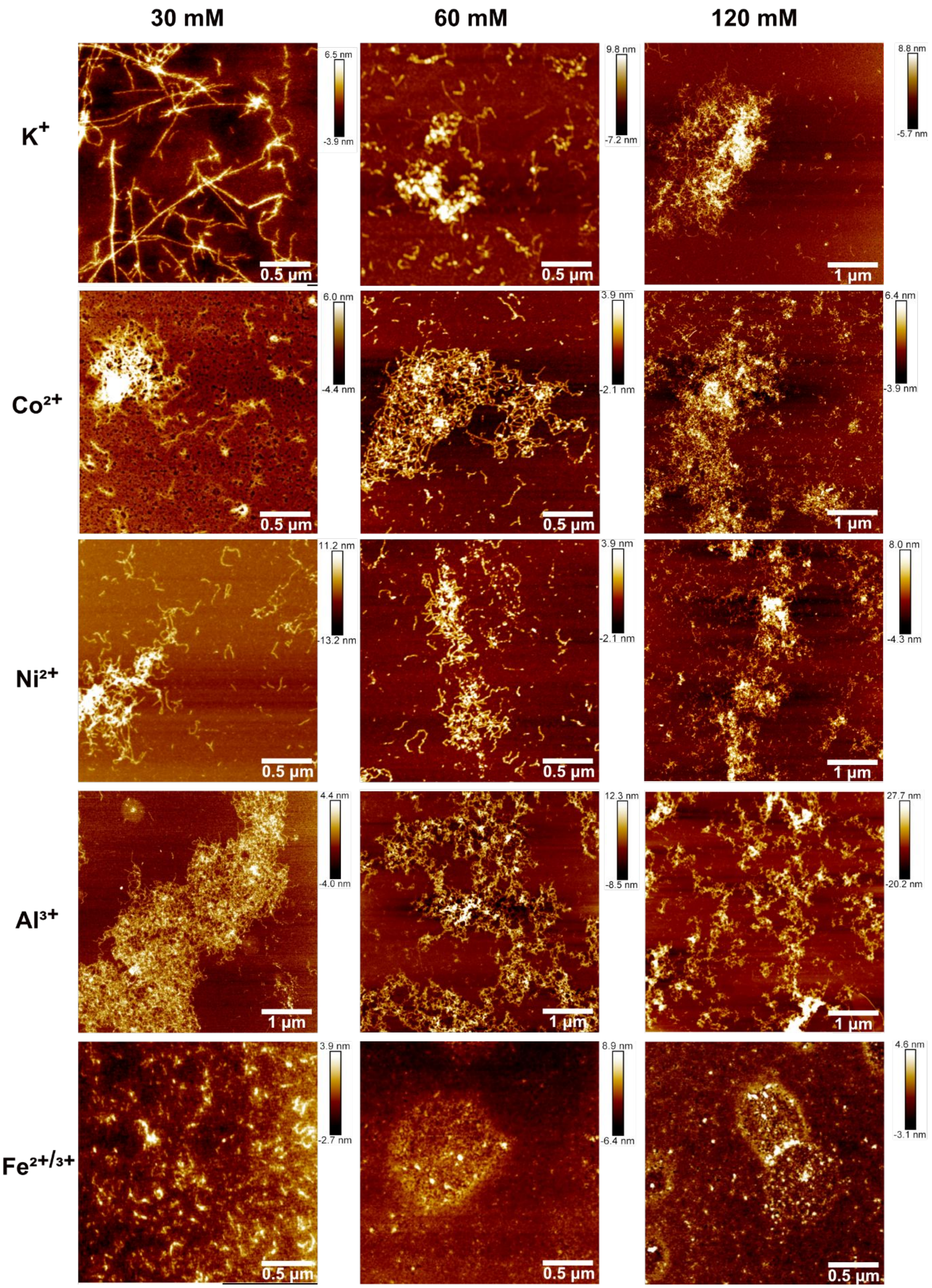

30 mM
60 mM
120 mM
K+
Co2+
Ni2+
Al3+
Fe2+/3+
0.5 µm
1 µm
6.5 nm
-3.9 nm
9.8 nm
-7.2 nm
8.8 nm
-5.7 nm
6.0 nm
-4.4 nm
3.9 nm
-2.1 nm
6.4 nm
-3.9 nm
11.2 nm
-13.2 nm
3.9 nm
-2.1 nm
8.0 nm
-4.3 nm
4.4 nm
-4.0 nm
12.3 nm
-8.5 nm
27.7 nm
-20.2 nm
3.9 nm
-2.7 nm
8.9 nm
-6.4 nm
4.6 nm
-3.1 nm

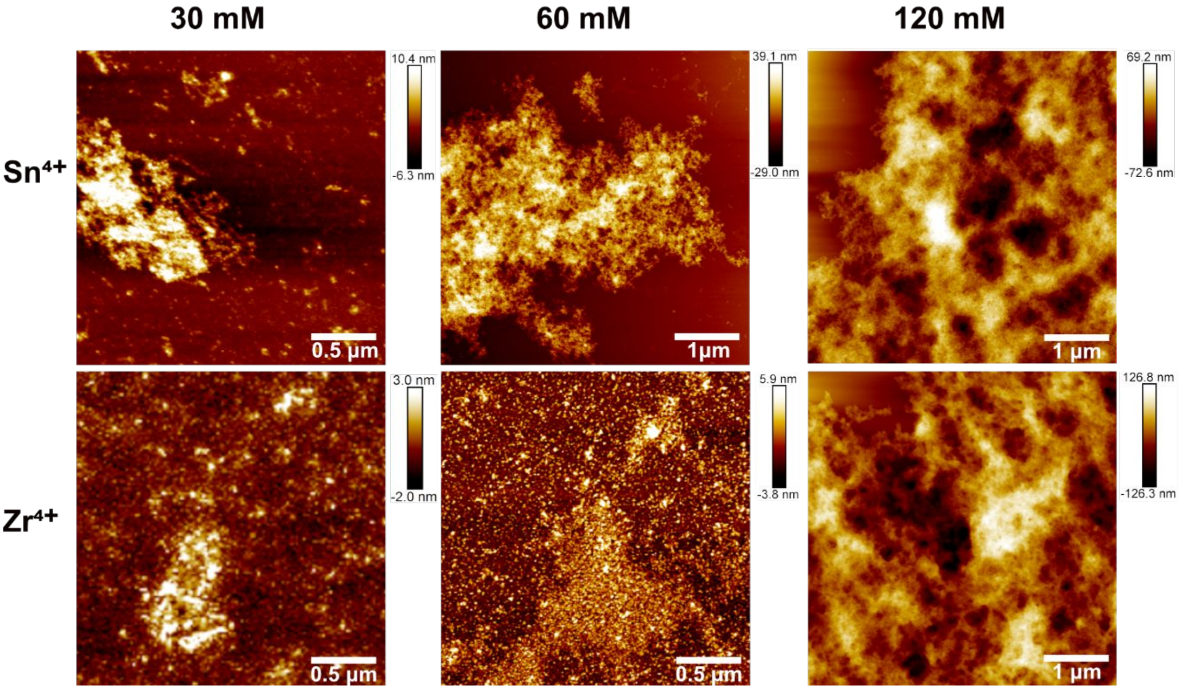

Figure S5. AFM images of PNFs generated with the presence of different metal ions.

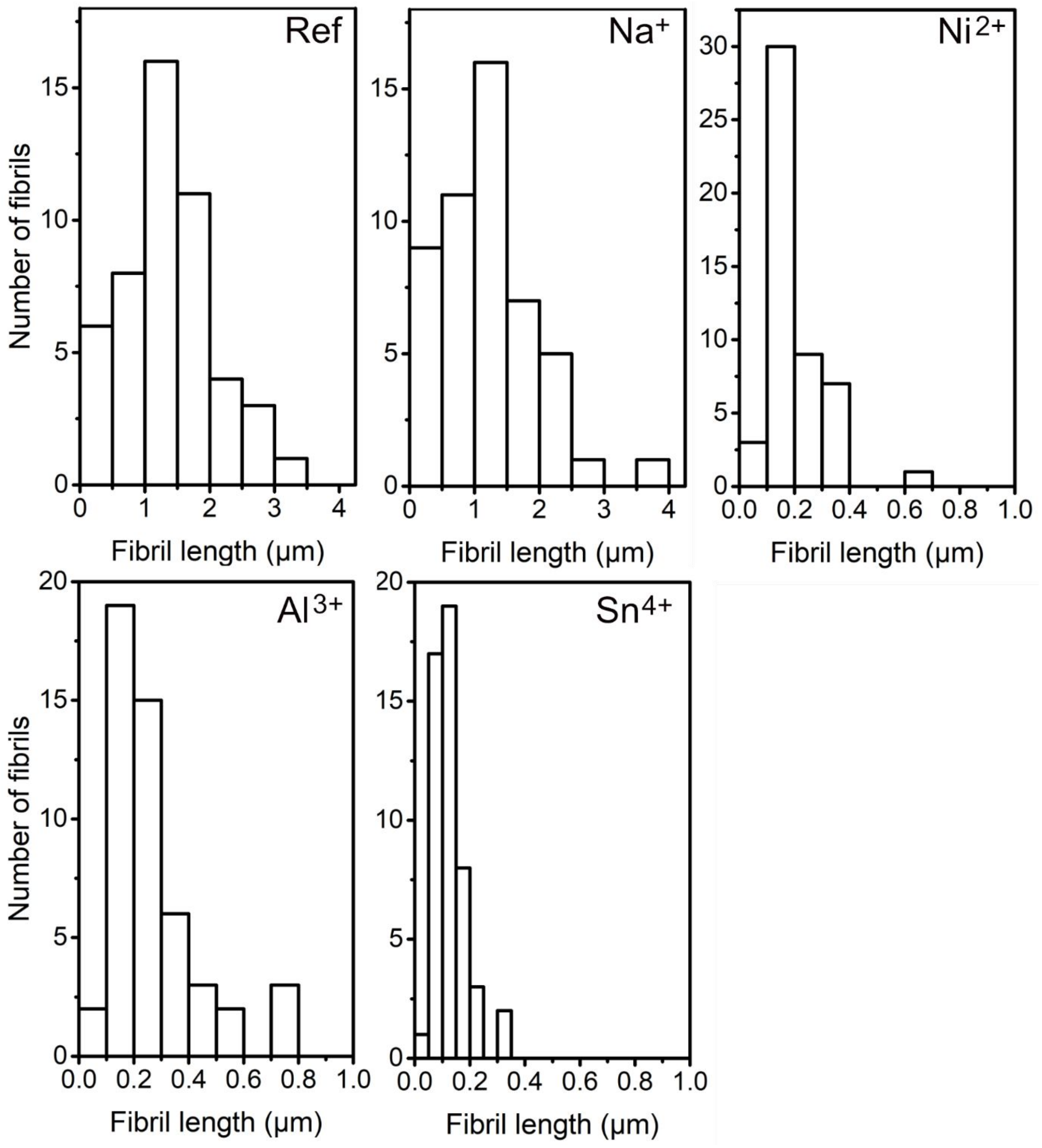

Figure S6. End-to-end length distribution of the PNFs formed in the reference, Na^{+}, Ni^{2+}, Al^{3+} and Sn^{4+} (30 mM) systems. The end-to-end distances of isolated fibrils (50 in total) in these systems were measured due to the complexity to measure accurately the contour length of curled fibrils.

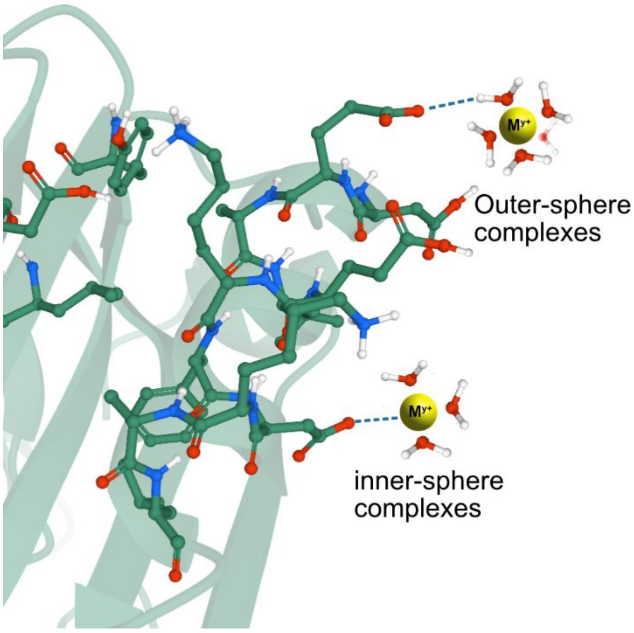

Figure S7. Suggested mechanism for the interaction between PNFs, metal ions and water molecules in the hydrogels. The proteins may interact with outer-hydration spheres (top) and/or within the inner-hydration spheres (bottom).

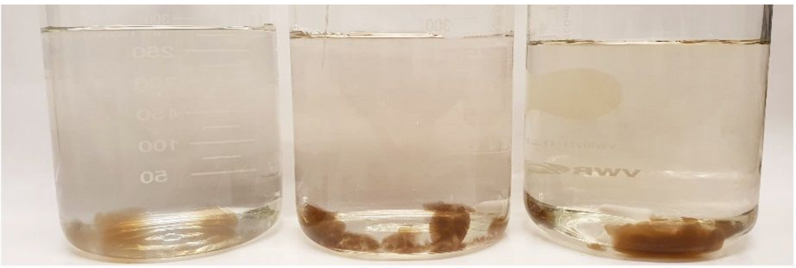

Figure S8. The PNF hydrogels containing Na^{+} (left)/Co^{2+} (middle)/Al^{3+} (right) immersed in MilliQ water at the beginning of the release experiment.

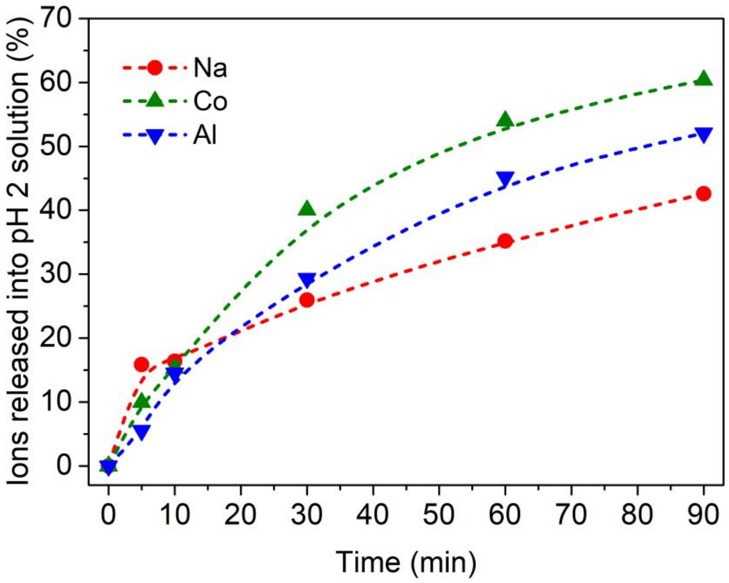

Figure S9. The kinetic study of Na^{+}, Co^{2+} and Al^{3+} ions released from PNF hydrogels in 0.01 M HCl solution. The hydrogels retained their shape and colour after 90 min immersion in the solution.

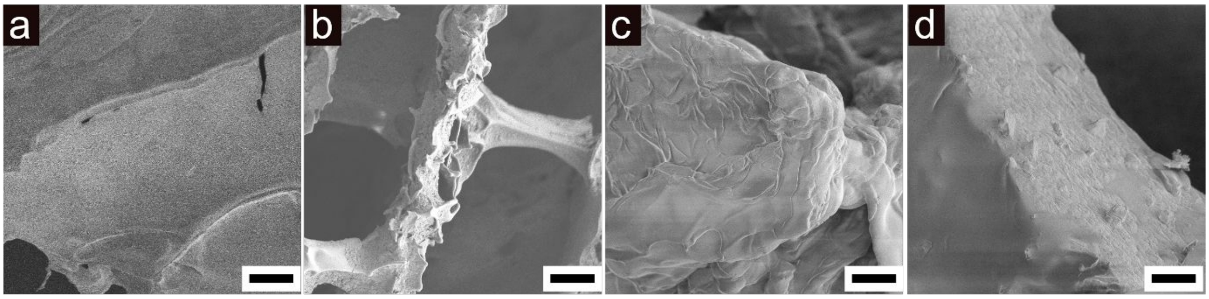

Figure S10. Microstructure of lyophilized PNF foams formed in the presence of 30 mM Al^{3+} (a), 60 mM Al^{3+} (b), 30 mM Sn^{4+} (c), 60 mM Sn^{4+}. The scale bars represents 10 μm.
